# Ursolic acid, a pentacyclic triterpene from *Ochrosia elliptica* Labill leaves and its role in the management of polycystic ovary syndrome (PCOS)

**DOI:** 10.1007/s10787-026-02158-w

**Published:** 2026-03-23

**Authors:** Shimaa R. Emam, Marwa A. Ibrahim, Fady Sayed Youssef, Marwa S. Khattab, Riham A. El-Shiekh, Rehab A. Ghandour, Heba F. Hozyen, Marwa Zakaria, Essam A. Abdel-Sattar, Samar M. Mouneir

**Affiliations:** 1 Department of Pharmacology, Faculty of Veterinary Medicine, Cairo University, Giza, 12211 Egypt; 2 Department of Biochemistry and Molecular Biology, Faculty of Veterinary Medicine, Cairo University, Giza, 12211 Egypt; 3 Department of Pathology, Faculty of Veterinary Medicine, Cairo University, Giza, 12211 Egypt; 4 Department of Pharmacognosy, Faculty of Pharmacy, Cairo University, El- Kasr El-Aini St, Cairo, 11562 Egypt; 5 Department of Physiology, Faculty of Veterinary Medicine, Cairo University, Giza, 12211 Egypt; 6 Animal Reproduction and Artificial Insemination Department, National Research Centre, Veterinary Research Institute, Tahrir Street, Dokki, Giza, 12622 Egypt; 7 Educational Central Lab, Women College, Ain Shams University, Cairo, Egypt

**Keywords:** Ursolic acid, Pentacyclic triterpene, Polycystic ovary syndrome (PCOS), Biochemical and hormonal biomarkers, Histopathology, Immunohistochemistry, Oxidative stress

## Abstract

Ursolic Acid (UA), a naturally occurring pentacyclic triterpene, was isolated from *Ochrosia elliptica*. Polycystic Ovary Syndrome (PCOS) a prevalent endocrine disorder characterized by hyperandrogenism, insulin resistance, and chronic inflammation. In this study, we investigate the role of UA in alleviating the symptoms of PCOS, focusing on its biochemical, hormonal, and histopathological effects in a rat model. Using adult female Wistar Albino rats, PCOS was induced through letrozole administration. The rats were then treated with UA at two different doses (25 and 50 mg/kg), alongside a control group and a standard ovulation-inducing medication, clomiphene citrate (1 mg/kg). Biochemical analyses showed that PCOS induction significantly increased serum malondialdehyde (MDA) levels by approximately 1.21-fold, while markedly reducing superoxide dismutase (SOD) and catalase (CAT) activities (*p* ≤ 0.05) by approximately 0.48-fold, relative to negative control. Treatment with UA (50 mg/kg) dose-dependently restored oxidative balance, reducing MDA (~ 0.82-fold) and elevating SOD (~ 2.39-fold) and CAT (~ 2.11-fold) activities toward PCOS values (*p* ≤ 0.05). Hormonally, PCOS rats exhibited elevated luteinizing hormone (LH) and testosterone levels by approximately 1.70-fold, compared to the negative control (*p* ≤ 0.05). Both doses of UA significantly lowered LH and testosterone, with the 50 mg/kg dose achieving reductions comparable to clomiphene citrate (*p* ≤ 0.05). Histopathological examination showed improved ovarian morphology with reduced cystic follicles and increased corpus lutea in UA-treated groups. Furthermore, UA downregulated key genes involved in steroidogenesis and oxidative stress response, suggesting a multifaceted mechanism of action. The findings highlight UA’s potential as a novel therapeutic option for managing PCOS symptoms, emphasizing the need for further research into its efficacy and safety in clinical applications.

## Introduction

Polycystic ovary syndrome (PCOS) is a heterogeneous endocrine disorder marked by aberrant gonadotropin production, hyperandrogenism, chronic anovulation, and polycystic ovarian morphology (Escobar-Morreale 2018). PCOS has a prevalence of 6 to 20% and typically appears in early adolescence (Siddiqui et al. 2022). PCOS manifests through symptoms related to elevated male sex hormones, including hirsutism, androgenetic alopecia, and acne. It is also linked to metabolic abnormalities, including insulin resistance (IR) (Dapas and Dunaif 2022; Zeng et al. 2020). The hyperactive hypothalamic-pituitary-gonadal (HPG) axis in PCOS patients leads to increased ovarian androgen production, primarily due to heightened activity of the CYP17A1 enzyme. Additionally, hyperinsulinemia lowers the synthesis of sex hormone-binding globulin (SHBG), resulting in higher levels of bioavailable androgens. While ovarian androgens have been implicated in the metabolic disturbances of PCOS, recent findings indicate that adrenal production of 11-oxygenated androgens may play a significant role, particularly as adrenal responsiveness to adrenocorticotropic hormone (ACTH) is heightened (Wang and Li 2023). The interaction between the ovaries, adipose tissue, and adrenal glands is crucial in the development of hyperandrogenism and insulin resistance (IR) in PCOS. Increased activity of aldo-keto reductase type 1C3 (AKR1C3) in adipose tissue converts androgens to more potent forms, particularly in obese individuals, leading to higher levels of 11-ketotestosterone. This relationship is further complicated by the risks of non-alcoholic fatty liver disease (NAFLD) and type 2 diabetes (T2D) in women with hyperandrogenism, irrespective of obesity (Chang and Dunaif 2021). Chronic low-grade inflammation is a significant contributor to PCOS, often linked to excess visceral adipose tissue. Factors like mitochondrial dysfunction and unhealthy dietary habits exacerbate oxidative stress, resulting in an imbalance of reactive oxygen species (ROS) (Dapas et al. 2020; Huddleston and Dokras 2022). While inflammation and oxidative stress can negatively impact reproductive health, they may also play roles in necessary physiological processes such as ovulation. Key signaling pathways, particularly the phosphoinositide-3 kinase/protein kinase B (PI3K/Akt) pathway, are essential for cell survival and glucose regulation (Dapas et al. 2020; Huddleston and Dokras 2022). In PCOS, altered luteinizing hormone (LH) activity disrupts insulin signaling, contributing to insulin resistance and impaired follicle development, and highlighting the complex nature of this syndrome (Dapas et al. 2020; Huddleston and Dokras 2022). To our knowledge, there is no clear standard for treating PCOS (Escobar-Morreale 2018; Teede et al. 2018). Because it cannot be totally cured, clinical treatment focuses on personalized drug therapy to address specific symptoms, notably hormone drugs. However, adverse effects are rather prevalent. Anti-androgen drugs, for example, diminish androgens’ biological effects through several pathways, reducing hirsutism and restoring ovulation (De Leo et al. 1998; Moghetti et al. 1994), but they may cause hepatotoxicity (Castelo-Branco and Del Pino 2009; Mendoza et al. 2014). The most often used PCOS medications are combined oral contraceptives that contain both progestins and/or estrogens (Mendoza et al. 2014). They control the menstrual cycle, prevent endometrial hyperplasia, and reduce hyperandrogenic symptoms. However, long-term use increases the risk of IR, venous thrombosis (Gronich et al. 2011), and menopausal symptoms (Belisle and Love 1986). Anti-estrogen medicines, such as clomiphene citrate (CC), are primary therapies for anovulatory infertility (Legro et al. 2013), although they might overstimulate the ovaries, resulting in multiple pregnancies (Kousta et al. 1997). Metformin dramatically improves IR and hyperandrogenism, and when combined with CC, it increases the ovulation-inducing effect (Pasquali 2014). Notably, metformin treatment primarily decreases testosterone levels but does not significantly affect 11-oxygenated androgens (Xie et al. 2021; Zhang et al. 2017). Although thiazolidinediones, which are likewise insulin sensitizers, might promote fluid retention and weight gain, their usage is not advised (Legro et al. 2013). Spironolactone treats hirsutism but can cause intermenstrual hemorrhage (Sabbadin et al. 2016; Tremblay 1986). Furthermore, the majority of these medicines are used off-label and have not been approved by the United States Food and Drug Administration (FDA) for PCOS therapy (Radosh 2009).

Despite obstacles in PCOS targeted therapy, natural products are gaining attention due to their broad therapeutic potential (Malik et al. 2024). These phytochemicals remarkably alter hormone regulation, and, being predominantly derived from natural sources, they generally reveal favorable safety profiles with low toxicity. Furthermore, most natural compounds have multifaceted actions, granting them reach outcomes that single-target medications cannot, especially in heterogeneous diseases. They can provide complementary therapies, increasing efficacy, decreasing side effects, and reducing reliance on prescription drugs when combined with traditional treatments (Ataabadi et al. 2017; Rudic et al. 2022; Yang et al. 2018).

**Fig. 1:**
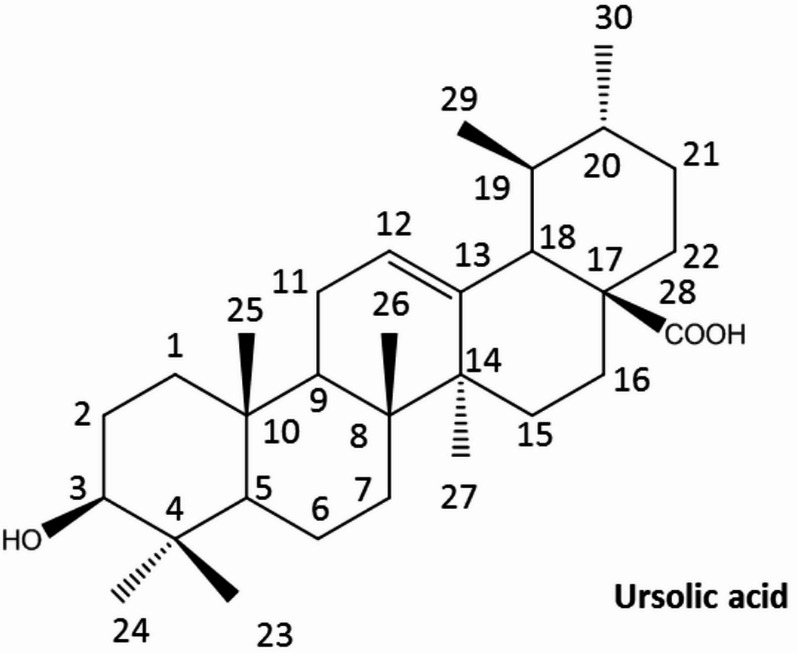
Chemical structure of ursolic acid (UA)

Ursolic acid (UA; 3β-hydroxy-urs-12-en-28-oic acid; Fig. 1) is a naturally occurring pentacyclic triterpene found in various fruits, herbs, and medicinal plants, including apple peels, rosemary, basil, and thyme. This bioactive compound has garnered significant attention due to its diverse range of pharmacological activities, such as anti-inflammatory, antioxidant, anticancer, and cardioprotective properties (Bakry et al. 2025). Structurally, UA is characterized by five fused carbon rings, which confer its unique biological activity and enable it to interact with multiple molecular targets within the body. Chemically, UA has the molecular formula C_30_H_48_O_3_​, featuring a hydroxyl group at the C-3 position, *β*- carboxyl group at C-17, a single double bond, and seven methyl groups within its ring system. These functional groups are critical for UA’s biological activity, facilitating hydrogen bonding, hydrophobic interactions, and metabolic transformations (Chen et al. 2015; Mlala et al. 2019). These effects are largely due to its ability to modulate key cellular pathways, such as inhibiting NF-κB and activating Nrf2/ARE, which help reduce oxidative stress and inflammation. Recent research also highlights UA’s potential in managing endocrine and metabolic disorders, such as polycystic ovary syndrome, which is characterized by high androgen levels, insulin resistance, inflammation, and oxidative imbalance (Khwaza and Aderibigbe 2024). Overall, UA’s complex chemistry and diverse pharmacological activities, along with its potential for structural modification, underline its significance in natural medicine and pharmaceutical development (Kornel et al. 2023; Labib et al. 2016).

The aim of the present study was to assess the effect of ursolic acid isolated from the leaves of *Ochrosia elliptica*, on letrozole-induced rat model of PCOS, with a focus on oxidative stress markers, hormonal profile, ovarian histopathology, and the expression of key genes involved in steroidogenesis and antioxidant response.

## Results

### Justification of the selected doses

We have reviewed the previously published in vivo studies of ursolic acid, commonly employed on different pharmacological modeles. Based on these studies of the using low (≈ 10 mg/kg) (Bang et al. 2023), moderate (25 mg/kg) (Furtado et al. 2008), high (50 mg/kg) (Bacanlı et al. 2018), and moderate–high (25–50 mg/kg) dose ranges (Ma et al. 2014, 2015; Wang et al. 2021; Xiang et al. 2012; Xu et al. 2018), we selected moderate–high (25–50 mg/kg) dose ranges. We omitted 10 mg/kg, which often shows limited efficacy, and are aiming to minimizing animal use while maintaining scientific rigor.

### Biochemical markers

**Fig. 2:**
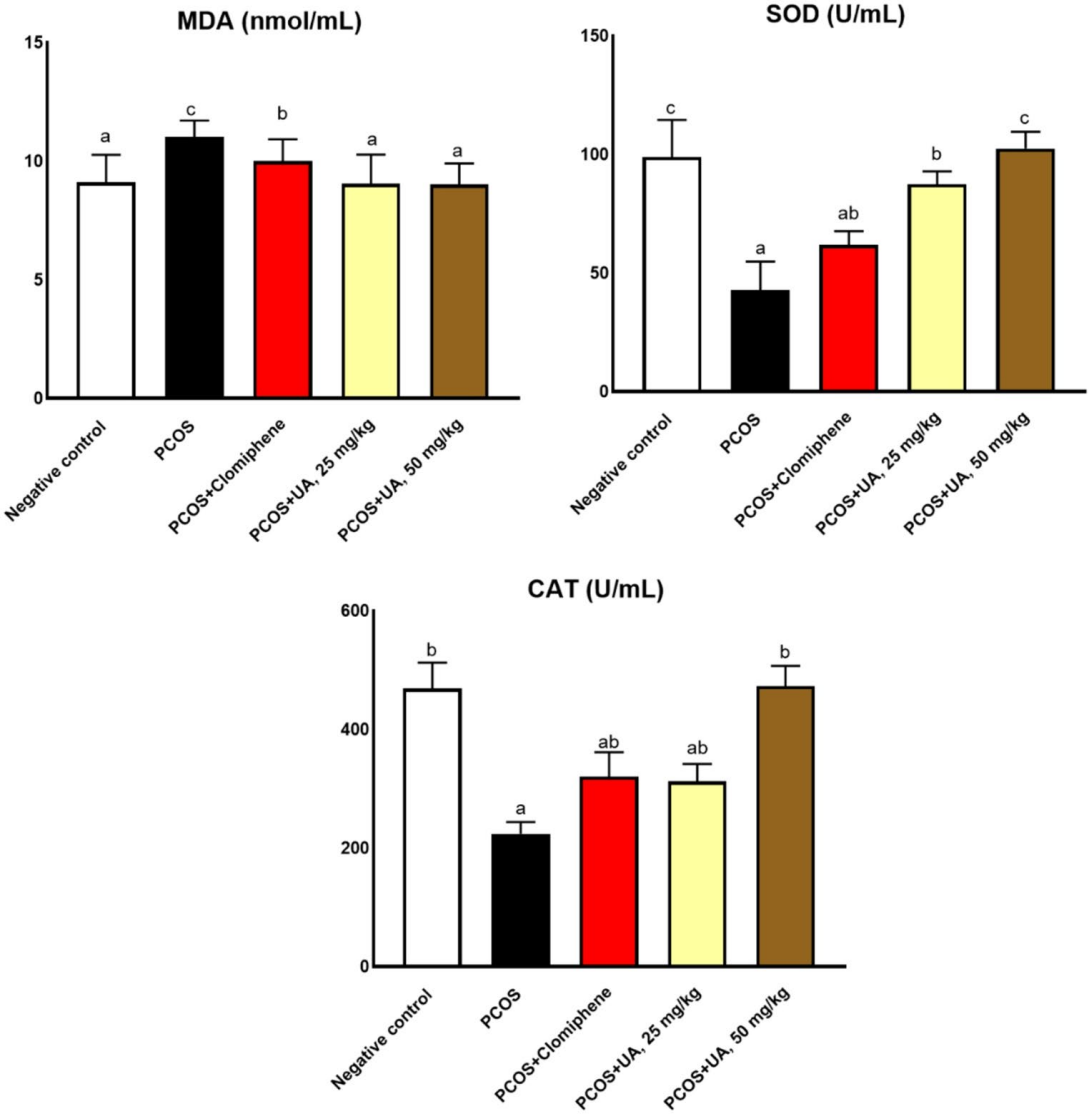
The antioxidant effects. Different superscripts indicate statistically significant differences at *P* ≤ 0.05. The values are represented as mean ± SE; n  = 7

**Fig. 3:**
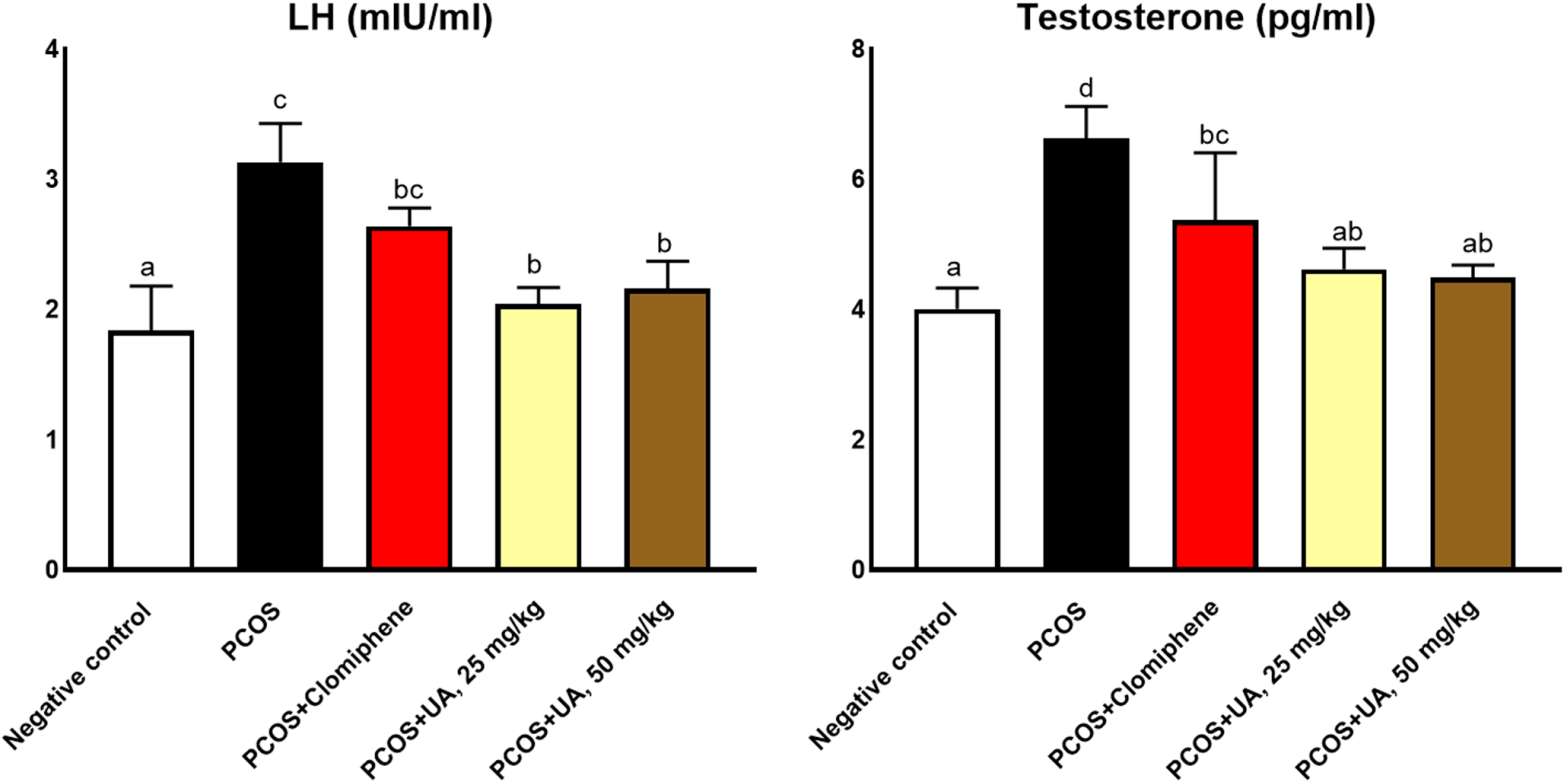
The hormonal effects. Different superscripts indicates statistically significant differences at *P* ≤ 0.05. The values are represented as mean ± SE; n = 7

The polycystic group showed significant increments in oxidative stress enzymes, MDA, and reduction in antioxidant enzymes (SOD and CAT). However, both doses of the UA significantly restored the adverse effects of PCOS (Fig. 2). Additionally, the polycystic group showed significant increments in hyperandrogenism hormones LH and testosterone. However, both doses of the UA significantly restored the adverse effects of PCOS (Fig. 3).

### Quantitative real-time PCR

**Fig. 4:**
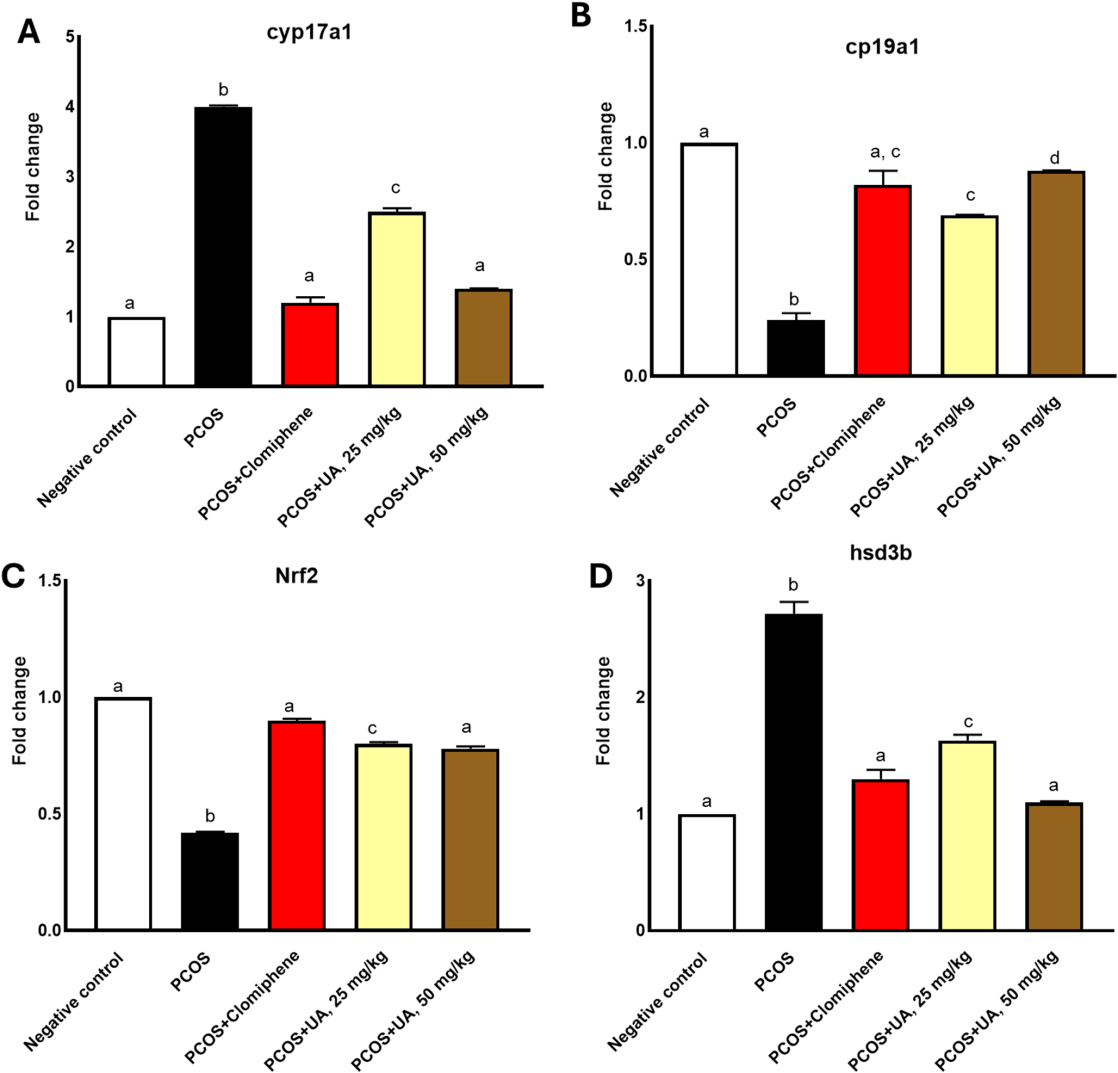
The gene expression results of **A** **CYP17A1**; **B** CYP19A1; **C** nrf-2; **D** hsp3d. Different superscripts indicate statistically significant differences at *P* ≤ 0.05. The values are represented as mean ± SE; n = 7

The polycystic group showed significant upregulations of **CYP17A1** and hsp3d, and downregulation of both the Nrf-2 and CYP19A1. However, both doses of the UA significantly ameliorated the adverse effects of PCOS (Fig. 4).

### Histopathological findings

**Fig. 5:**
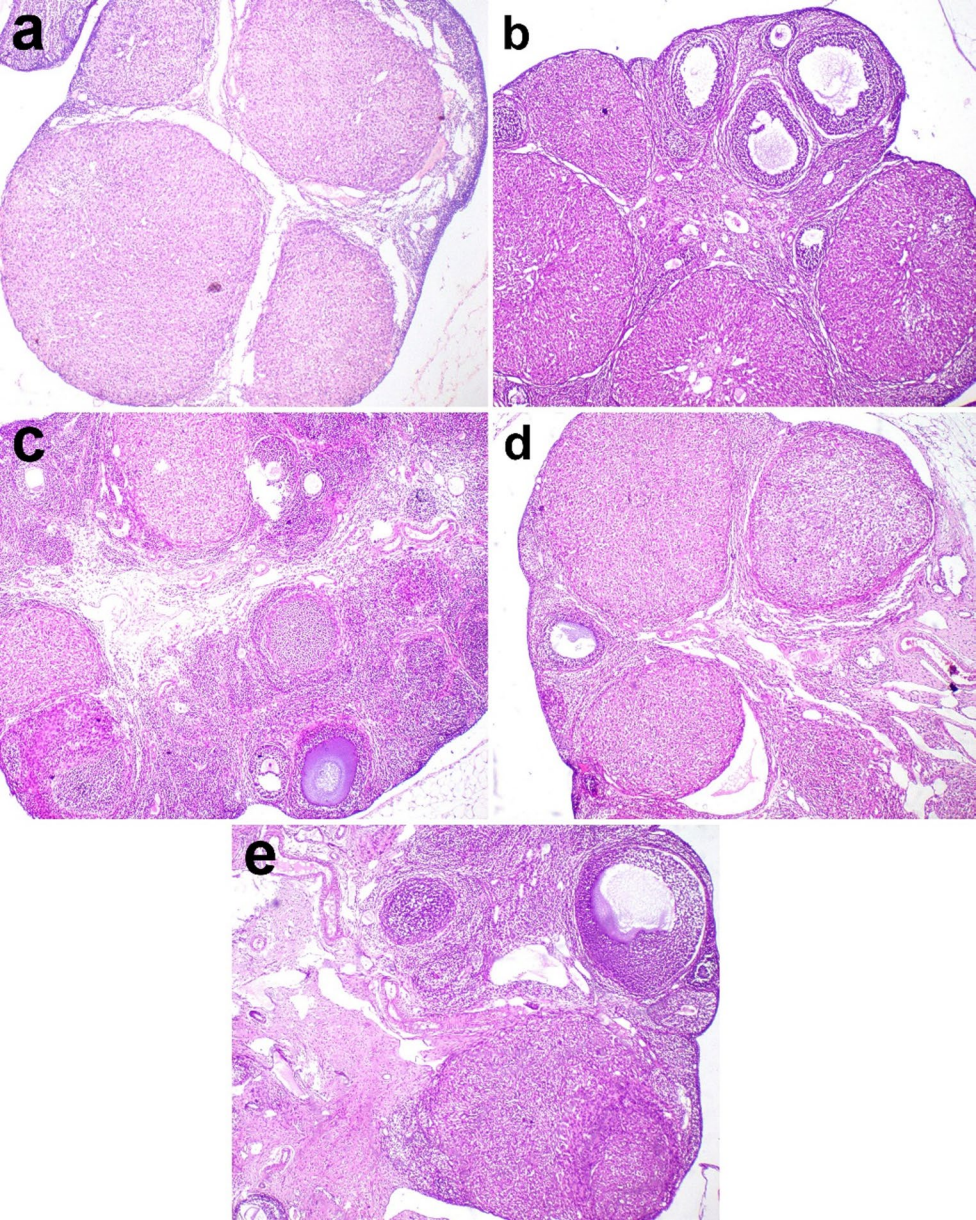
Histopathological micrographs of ovaries in rats of various groups. **a** multiple corpora luti in the ovary of G1, **b** many follicles with large antrum in G2, **c** few follicles and many corpora luti in G3, and **d** G4 and **e** a moderate number of follicles and corpora luti in G5. Hematoxylin and eosin stain X 100

Ovarian histopathology showed corpus luti and developmental stages of different sized follicles with multiple layers of granuolsa cells (Fig. 5a**)**. Microscopy of G2 (letrazole group) ovary revealed many follicles with antrum lined with 2–3 layers of granulosa cells in addition to subcapsular large cystic follicles (Fig. 5b). Microscopy of ovary in G3 (clomiphene citrate) showed few follicles in variable stages of development with a decrease in number of cystic follicles and corpora lutea (Fig. 5c). In G4 and G5, the number of cystic follicles decreased remarkably and multiple corpus luti were observed (Fig. 5d, e).

### Immunohistochemical findings

**Fig. 6:**
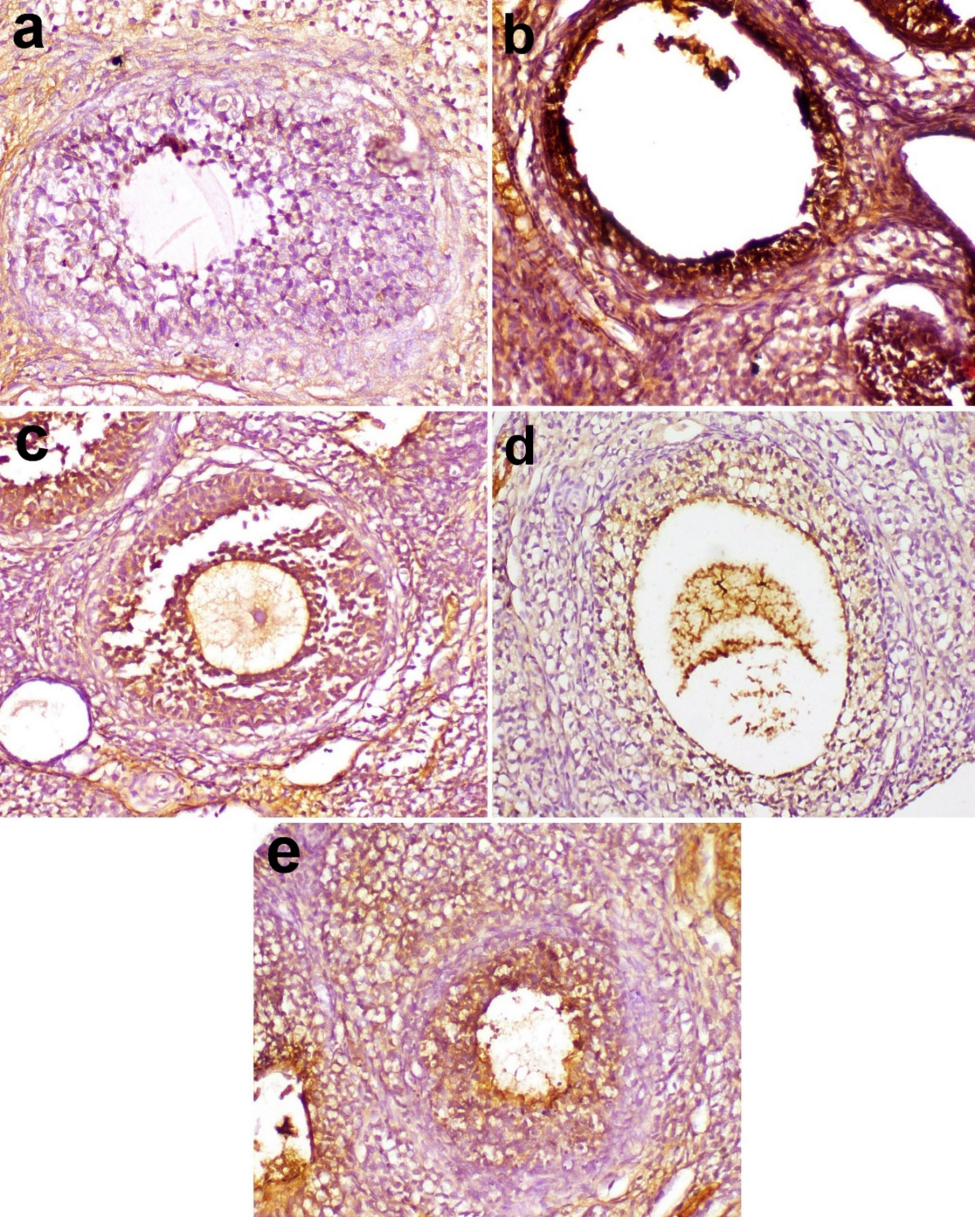
Caspase-3 immunoexpression in ovaries of rats in various groups. **a** moderate expression in ovary in G1, **b** severe expression in follicles with a large antrum in G2, **c** moderate expression in G3, **d** mild to moderate expression in G4, and **e** mild immunoexpression in G5. Immunoperoxidase X 200

**Fig. 7:**
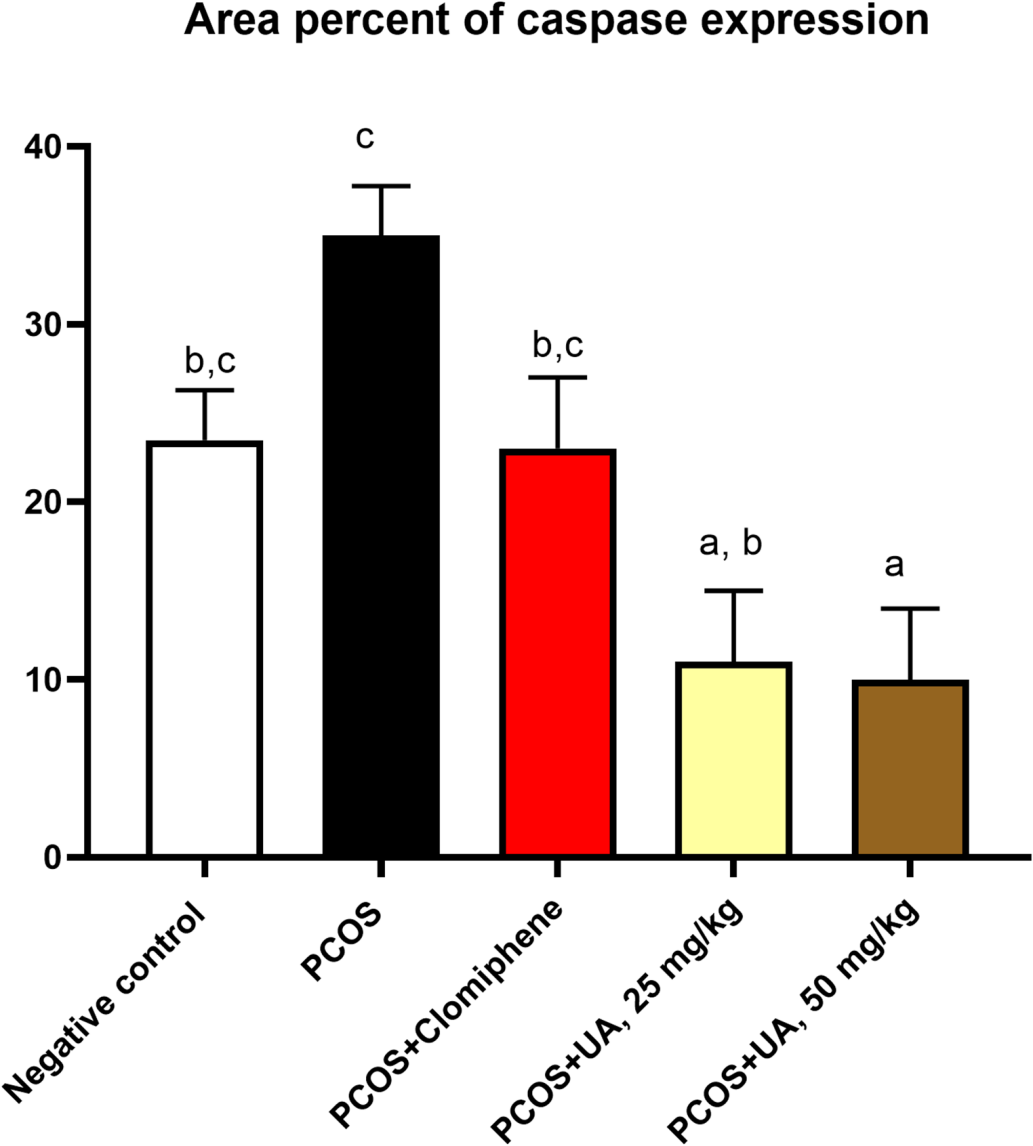
The percentage of the area of caspase-3 immunoexpression. Columns denote the mean value ±standard error. Columns having dissimilar lowercase letters are considered significant at *P* value < 0.05. ANOVA test followed by Duncan test

The expression of caspase-3 in ovaries was seen in follicular granulosa cells (Figs. 6 and 7). It was moderate in the ovaries of rats in the control group (Fig. 6a), severely increased in moderate and large sized follicles having thin lining of granulosa cells and large antrum in G2 group (Fig. 6b). In G3, caspase-3 expression was comparable to that seen in G1 (Fig. 6c). In G4 and G5, the expression was remarkably decreased in relation to the rest of groups in the ovaries of rats (Fig. 6d, e).

## Discussion

Ursolic acid (UA, 3 β-hydroxy-urs-12-en-28-oic acid) is a natural terpene compound known for its diverse pharmaceutical properties, including anti-inflammatory, anticancer, antidiabetic, antioxidant, and antibacterial effects (Mlala et al. 2019; Woźniak et al. 2015). Additionally, UA is recognized for its protective effects on the lungs, kidneys, liver, and brain, as well as its anabolic effects on skeletal muscles and its ability to prevent bone density loss related to osteoporosis. Furthermore, ursolic acid exhibits antimicrobial features against various strains of bacteria, HIV, HCV viruses, and the Plasmodium protozoa responsible for malaria.

The findings of this study show that ursolic acid (UA) effectively reduces oxidative stress and hormonal imbalances often seen in polycystic ovary syndrome (PCOS). Increased oxidative stress plays a crucial role in poor follicle development and disrupted steroid hormone production in PCOS. This is supported by higher levels of malondialdehyde (MDA) and lower activities of antioxidant enzymes like superoxide dismutase (SOD) and catalase (CAT). Previous studies have also reported increased lipid peroxidation and a weakened antioxidant system in PCOS models (Cheng and He 2022). In this context, UA has shown significant antioxidant effects by lowering MDA levels and boosting SOD and CAT activities (Zhao et al., 2023), with notable effectiveness at a dose of 50 mg/kg (Bakry et al. 2025).

The decrease in luteinizing hormone (LH) levels after UA treatment suggests a partial improvement in the regulatory function of the hypothalamus and pituitary gland, which is often lacking in PCOS. The observed hormonal improvements may also be partly due to UA antioxidant and anti-inflammatory properties, given the well-known relationship between oxidative stress, inflammation, and hormone production in PCOS. Together, this evidence indicates that UA reduction of oxidative stress may help restore normal reproductive hormone levels by improving the ovarian environment and decreasing oxidative and inflammatory damage to ovarian tissue.

At the same time, the PCOS model showed significant hormonal disruptions, characterized by high serum levels of LH and testosterone. This reflects issues in the hypothalamic-pituitary-gonadal (HPG) axis and ovarian excess androgen production. These hormonal changes stem mainly from irregular gonadotropin-releasing hormone (GnRH) pulses, which lead to excessive LH release and overstimulation of ovarian theca cells, causing increased androgen production (Lőrincz et al., 2024). Supporting these mechanisms, UA treatment significantly reduced circulating LH and testosterone levels, achieving results similar to standard medications at a dose of 50 mg/kg (Besasie et al. 2024). The drop in LH levels after UA treatment may indicate some recovery of the hypothalamic-pituitary control, which is often impaired in PCOS. Additionally, the noted hormonal improvements may be partly driven by UA antioxidant properties, considering the link between oxidative stress, neuroendocrine dysfunction, and hormone production in PCOS. The drop in testosterone levels aligns with strong evidence identifying abnormal theca cell function as a key factor in PCOS-related excess androgen levels (Rosenfield & Ehrmann, 2016). However, further detailed investigations are needed to clarify the specific molecular pathways through which UA influences the hypothalamic-pituitary-ovarian axis.

Overall, these results suggest that UA’s reduction of oxidative stress may indirectly support the normalization of reproductive hormone levels by improving the ovarian follicular environment and reducing oxidative damage in ovarian tissue.

The anti-inflammatory and pro-apoptotic properties of UA, as demonstrated in psoriasis research, suggest a promising application for Polycystic Ovary Syndrome (PCOS), a condition also associated with chronic inflammation and hyperproliferation (Bielecka et al. 2024). In psoriasis models, UA was effective in reducing inflammatory cytokine production, particularly IL-6 and IL-8, and in decreasing the expression of psoriatic biomarkers (S100A7, S100A8, and S100A9) (Bielecka et al. 2024). These results support the potential for UA to mitigate similar inflammatory pathways in PCOS, which is also characterized by elevated levels of IL-6 and other pro-inflammatory cytokines. The previous findings further highlight UA’s capacity to induce non-inflammatory apoptosis selectively in keratinocytes without adversely affecting immune cells. This specificity may be beneficial in PCOS, as it could limit follicular hyperproliferation and promote cellular turnover in the ovaries while minimizing systemic immune effects (Bielecka et al. 2024).

In addition to its apoptotic and anti-inflammatory effects, UA shows potential for modulating androgen receptor activity, which is relevant to PCOS, where hyperandrogenism plays a significant role in disease pathology. In prostate cancer studies (Besasie et al. 2024), UA has been observed to impact androgen receptor pathways, potentially reducing androgen-driven cellular proliferation. This mechanism may have beneficial applications in PCOS by targeting androgenic signaling, which contributes to ovarian hyperplasia and anovulation.

Through a combined effect (curcumin and UA) on androgen receptors, NF-κB inhibition, and caspase-mediated apoptosis, UA could address key aspects of PCOS, such as inflammation, follicular overgrowth, and hormonal imbalance. This multi-targeted approach reinforces the potential of UA as a novel therapeutic option for managing PCOS symptoms, especially hyperandrogenism and its effects on ovarian function (Besasie et al. 2024).

UA possesses several anti-inflammatory properties that may be particularly relevant for treating PCOS, a condition in which inflammation contributes to hormonal imbalances and metabolic complications. Inflammation plays a critical role in PCOS pathophysiology, driving insulin resistance, hyperandrogenism, and follicular dysregulation. By suppressing key inflammatory pathways, UA may help reduce the underlying inflammatory environment contributing to PCOS symptoms (Bakry et al. 2025).

The mechanism of action of UA involves downregulation of NF-κB and the genes it regulates, including those encoding pro-inflammatory cytokines and enzymes such as cyclooxygenase-2 (COX-2) and lipoxygenase. This suppression can be beneficial in PCOS by potentially reducing cytokine-induced disruptions in ovarian function and mitigating the oxidative stress linked to PCOS-associated insulin resistance. Additionally, UA’s ability to reduce intracellular reactive oxygen species (ROS) and inhibit lipid peroxidation may protect ovarian cells from oxidative damage, which can otherwise lead to ovarian dysfunction (Cheng and He 2022; Desai et al. 2014).

UA’s structural characteristics as a pentacyclic triterpene also allow it to impact extracellular and intracellular pathways related to inflammation and apoptosis, which could improve ovarian health by regulating cell proliferation and reducing inflammation-induced follicular cyst formation (Khwaza and Aderibigbe 2024).

In addition, previous studies reported the use of UA as cardioprotective against doxorubicin-induced cardiac toxicity. UA improved heart function, increased nitric oxide (NO) production, reduced oxidative stress, and prevented cell apoptosis through mechanisms involving eNOS and AKT pathway activation. These effects, particularly UA’s activation of eNOS and enhanced NO levels, may benefit PCOS by improving ovarian blood flow, supporting follicular health, and reducing cyst formation (Mu et al. 2019).

Furthermore, UA inhibited the activity of topoisomerase II in a molecular docking study, an enzyme involved in DNA replication and cell cycle regulation. Abnormal proliferation of ovarian theca cells in PCOS is linked to elevated androgen levels, and inhibiting topoisomerase II could help mitigate this hyperproliferation. By reducing the activity of topoisomerase II, UA might not only address the inflammation associated with PCOS but also play a role in normalizing cell growth and differentiation in ovarian tissues (Srinivasan et al. 2020).

The findings of this study show that ursolic acid (UA) effectively reduces oxidative stress and hormonal imbalances often seen in polycystic ovary syndrome (PCOS). Increased oxidative stress plays a crucial role in poor follicle development and disrupted steroid hormone production in PCOS. This is supported by higher levels of malondialdehyde (MDA) and lower activities of antioxidant enzymes like superoxide dismutase (SOD) and catalase (CAT). Previous studies have also reported increased lipid peroxidation and a weakened antioxidant system in PCOS models (Cheng and He 2022; El-Komy et al., 2023; Shah et al., 2022). In this context, UA has shown significant antioxidant effects by lowering MDA levels and boosting SOD and CAT activities (Zhao et al., 2023), with notable effectiveness at a dose of 50 mg/kg (Bakry et al. 2025).

The decrease in luteinizing hormone (LH) levels after UA treatment suggests a partial improvement in the regulatory function of the hypothalamus and pituitary gland, which is often lacking in PCOS. The observed hormonal improvements may also be partly due to UA antioxidant and anti-inflammatory properties, given the well-known relationship between oxidative stress, inflammation, and hormone production in PCOS. Together, this evidence indicates that UA reduction of oxidative stress may help restore normal reproductive hormone levels by improving the ovarian environment and decreasing oxidative and inflammatory damage to ovarian tissue.

At the same time, the PCOS model showed significant hormonal disruptions, characterized by high serum levels of LH and testosterone. This reflects issues in the hypothalamic-pituitary-gonadal (HPG) axis and ovarian excess androgen production. These hormonal changes stem mainly from irregular gonadotropin-releasing hormone (GnRH) pulses, which lead to excessive LH release and overstimulation of ovarian theca cells, causing increased androgen production (Lőrincz et al., 2024). Supporting these mechanisms, UA treatment significantly reduced circulating LH and testosterone levels, achieving results similar to standard medications at a dose of 50 mg/kg (Besasie et al. 2024). The drop in LH levels after UA treatment may indicate some recovery of the hypothalamic-pituitary control, which is often impaired in PCOS. Additionally, the noted hormonal improvements may be partly driven by UA antioxidant properties, considering the link between oxidative stress, neuroendocrine dysfunction, and hormone production in PCOS. The drop in testosterone levels aligns with strong evidence identifying abnormal theca cell function as a key factor in PCOS-related excess androgen levels (Rosenfield & Ehrmann, 2016). However, further detailed investigations are needed to clarify the specific molecular pathways through which UA influences the hypothalamic-pituitary-ovarian axis.

### Study limitations

Despite the promising findings, several limitations of the present study should be considered. First, the study was conducted exclusively in a letrozole-induced rat model of PCOS, which, although well established, may not fully replicate the heterogeneity and complexity of PCOS in humans. Therefore, extrapolation of these results to clinical practice should be translated with caution. Second, the study was evaluated using two doses only and over a relatively short treatment period, and long-term efficacy, safety, and dose–response relationships were not assessed. Third, additional metabolic indices such as insulin resistance, glucose tolerance, and lipid profile were not investigated. No behavioural tests were performed, so functional outcomes remain speculative. Moreover, the molecular mechanisms underlying ursolic acid’s effects on the hypothalamic–pituitary–ovarian axis were not explored in depth. Finally, pharmacokinetic and bioavailability data for ursolic acid were not determined, which may influence its translational potential. Future studies addressing these limitations are warranted to further validate and extend the current findings.

## Conclusions

The current study demonstrates that UA possesses promising therapeutic potential for managing PCOS. The results indicate that UA mitigates oxidative stress, hormonal imbalances, and histopathological alterations associated with PCOS in a rat model. By targeting multiple pathways, including inflammation and steroidogenesis, UA offers a comprehensive approach to addressing the complex nature of this syndrome. The favorable safety profile of UA, derived from natural sources, enhances its appeal as a complementary therapy alongside conventional treatments. Given the increasing prevalence of PCOS and its associated complications; however, these findings are preclinical and further mechanistic and clinical investigations are required before translational application can be considered. Future studies should aim to establish optimal dosing regimens, long-term effects, and potential synergistic interactions with existing treatments to fully realize the therapeutic benefits of UA in PCOS management.

## Materials and methods

### General

NMR analysis (^1^H-NMR: 400 MHz and ^13^C-NMR: 100 MHz) was performed on a Bruker High Performance Avance III FT-NMR spectrometer (Bremen, Germany) using TMS as internal standard. Chemical shift values were recorded in δ ppm.

### Plant material and preparation of extracts

The leaves of *Ochrosia elliptica* Labill. were collected from El-Orman Botanical Garden, Giza, Egypt, in April 2021. The plant material was kindly identified by Mrs. Therese Labib, Consultant of Plant Taxonomy at the Ministry of Agriculture and the Former Director of El-Orman Botanical Garden. Voucher specimen NO. (28.12.2021) is kept at the Herbarium of the Department of Pharmacognosy, Faculty of Pharmacy, Cairo University.

Five kilograms of the air-dried powdered leaves were extracted with ethanol 95% (3 × 6.5 L) by cold maceration. The ethanolic extract was combined and evaporated under reduced pressure to dryness (700 g). An aliquot of the dry residue (500 g) was then suspended in water (1 L) and partitioned successively with petroleum ether (5 × 1 L), methylene chloride (5 × 1 L), and *n*-butanol saturated with water (5 × 1 L). The solvents were evaporated under reduced pressure to give petroleum ether fraction (148 g), methylene chloride fraction (59 g), *n*-butanol fraction (140 g) and remaining water fraction (147 g).

### Isolation of ursolic acid

25 g of the methylene chloride fraction were subjected to a flash column chromatography (25 × 15 cm), packed with 600 g silica gel 60 (270–400 mesh). Gradient elution was performed using mixture of ethyl acetate-methanol-ammonium hydroxide (98:2:0.1) with increasing the polarity up to 90:10:0.1 and ending with 100% methanol. Five main fractions were collected. Fraction IV (5 g) was further chromatographed on a silica gel 60 (70–230 mesh) column (25 × 2.5 cm) using *n*-hexane-ethyl acetate (97:3 v/v) as eluent to yield 2.8 g of a white powder (compound C_1_). Compound C_1_ was identified as ursolic acid (UA) using NMR analysis (^1^H- and ^13^C-NMR) and compared to reported spectral data (El-Shiekh et al. 2021).

^**1**^**H-NMR** ppm (400 MHz, DMSO-d_6_ ): 5.16 (1 H, br s, H-12), 4.31 (1 H, br s, OH), 3.00 (1 H, dd, J = 12, 6.4 Hz, H-3), 2.73 (1 H, d, J = 16 Hz, H-18), 1.04 (3 H, s, Me-23), 0.92 (3 H, s, Me-27), 0.90 (3 H, s, Me-26), 0.85 (3 H, s, Me-24), 0.82 (3 H, d, J = 5 Hz, Me-30), 0.75 (3 H, d, J = 4 Hz, Me-29), 0.72 (3 H, s, Me-25).

^**13**^**C-NMR** ppm (100 MHz, DMSO-d_6_): 39.47 (C-1), 28.22 (C-2),76.64 (C-3), 38.42 (C-4), 54.75 (C-5), 17.97 (C-6), 32.81 (C-7), 40.10 (C-8), 47.06 (C-9), 36.57 (C-10), 23.23 (C-11), 124.46 (C-12), 138.09 (C-13), 41.61 (C-14), 27.51 (C-15), 23.79 (C-16), 46.79 (C-17), 52.35 (C-18), 39.89 (C-19), 39.89 (C-20), 30.12 (C-21), 36.49 (C-22), 28.22 (C-23), 15.19 (C-24), 15.99 (C-25), 16.90 (C-26), 23.23 (C-27), 178.30 (C-28), 16.99 (C-29), 21.05 (C-30).

### Animals

The experimental protocol was approved by the Institutional Animal Care and Use Committee at Cairo University (CU-IACUC; Vet CU1220251277). The study utilized adult female Wistar Albino rats, weighing between 160 and 200 g, all of which were virgin and displayed regular estrous cycles. These animals were obtained from the Laboratory Animal Colony in Helwan, Egypt, and housed within the animal facility at the Faculty of Veterinary Medicine, Cairo University. Prior to the commencement of the experiment, the rats underwent a two-week acclimatization period. During the study, they were maintained in standard polypropylene cages under controlled environmental conditions, including a temperature of (22 ± 3) °C, humidity at (55 ± 5) %, and a 12-h light/dark cycle. The rats were provided with a standard diet and had free access to water.

### Experimental design

Thirty-five female rats were randomly assigned to five groups, with seven rats in each group (Ali et al. 2021). Animals were randomly allocated to experimental groups using a simple randomization procedure to minimize selection bias. Group assignment was performed prior to the start of treatment, and all animals had an equal probability of being assigned to any the experimental groups. **Group 1** served as the negative control and received a daily oral dose of 1 mL vehicle (0.5% carboxymethyl cellulose (CMC) for 58 days via gavage. Polycystic Ovary Syndrome (PCOS) was induced following a well-established rat model (Azouz et al. 2021; Mannerås et al. 2007). **Groups 2** to **5** were administered letrozole (LTZ) at a dosage of 1 mg/kg dissolved in 0.5% CMC daily for 21 days to induce PCOS. After this induction phase, the groups underwent a 28-day treatment regimen with various samples: **Group 2** (PCOS group) received only the vehicle; **Group 3** was treated with clomiphene citrate (1 mg/kg in 0.5% CMC), a standard ovulation induction medication (Fertyl-Super tablets from Ar-Ex Laboratories Private Limited, Mumbai) (Kar and Sanchita 2015). **Groups 4** and **5** received UA at low (25 mg/kg) and high (50 mg/kg) doses, respectively (Ma et al. 2014, 2015; Xiang et al. 2012; Xu et al. 2018).

Starting on the sixth day of treatment, daily vaginal smears were collected from all rats to monitor ovulation. An abnormal estrous cycle, characterized by a prolonged diestrus phase, is a hallmark symptom of PCOS (Srinivasan et al. 2020). On the 50th day of the study, all rats were anesthetized using intraperitoneal thiopental sodium (40 mg/kg), and duplicate blood samples were collected into sodium heparin tubes for plasma separation and gel separator tubes for serum collection. Serum was separated through centrifugation at 3000*g* at 4 °C for 10 min, followed by biochemical analyses.

Following blood collection, the animals were sacrificed, and their ovaries and uteri were excised, cleaned of adipose tissue, weighed, and divided into triplicates. Two sets were stored at − 80 °C for quantitative real-time reverse transcriptase-polymerase chain reaction (qRT-PCR) and antioxidant assays, while another set of ovaries was fixed in 10% neutral buffered formalin for histopathological examination. The relative weights of the ovaries and uteri were calculated as the ratio of organ weight (mg) to body weight (g) (Kar and Sanchita 2015; Rajan and Balaji 2017).

### Biochemical markers

#### Assessment of oxidative stress

The oxidant and antioxidant biomarkers were evaluated in rat serum using kits purchased from Biodiagnostic, Egypt.

##### Malondialdehyde (MDA)

Malondialdehyde (MDA) levels, used as an indicator of lipid peroxidation, were determined following the method described by Ohkawa et al. (1979) (Ohkawa et al. 1979). This test is based on the reaction between thiobarbituric acid and MDA in an acidic environment, carried out at 95 °C for 30 min.

##### Superoxide dismutase enzyme (SOD) activity

SOD activity in rat serum was assessed using the method outlined by Nishikimi et al. (1972) (Nishikimi et al. 1972). The assay relies on the SOD enzyme’s ability to inhibit the reduction of nitro blue tetrazolium dye, which is mediated by phenazine methosulphate.

##### Catalase enzyme activity (CAT)

CAT activity was measured based on its reaction with hydrogen peroxide (H2O2) as described by Aebi (1974) (Aebi 1974). After one minute, the reaction was halted with a catalase inhibitor. In the presence of horseradish peroxidase (HRP), the remaining H2O2 reacts with a chromogen (3,5-dichloro-2-hydroxybenzene sulfonic acid) and 4-aminophenazone, forming a chromophore. The color intensity of the resulting product is inversely related to the catalase activity in the original sample.

The findings of this study show that ursolic acid (UA) effectively reduces oxidative stress and hormonal imbalances often seen in polycystic ovary syndrome (PCOS). Increased oxidative stress plays a crucial role in poor follicle development and disrupted steroid hormone production in PCOS. This is supported by higher levels of malondialdehyde (MDA) and lower activities of antioxidant enzymes like superoxide dismutase (SOD) and catalase (CAT). Previous studies have also reported increased lipid peroxidation and a weakened antioxidant system in PCOS models (Cheng and He 2022; El-Komy et al., 2023; Shah et al., 2022). In this context, UA has shown significant antioxidant effects by lowering MDA levels and boosting SOD and CAT activities (Zhao et al., 2023), with notable effectiveness at a dose of 50 mg/kg (Bakry et al. 2025).

The decrease in luteinizing hormone (LH) levels after UA treatment suggests a partial improvement in the regulatory function of the hypothalamus and pituitary gland, which is often lacking in PCOS. The observed hormonal improvements may also be partly due to UA antioxidant and anti-inflammatory properties, given the well-known relationship between oxidative stress, inflammation, and hormone production in PCOS.Together, this evidence indicates that UA reduction of oxidative stress may help restore normal reproductive hormone levels by improving the ovarian environment and decreasing oxidative and inflammatory damage to ovarian tissue.

At the same time, the PCOS model showed significant hormonal disruptions, characterized by high serum levels of LH and testosterone. This reflects issues in the hypothalamic-pituitary-gonadal (HPG) axis and ovarian excess androgen production. These hormonal changes stem mainly from irregular gonadotropin-releasing hormone (GnRH) pulses, which lead to excessive LH release and overstimulation of ovarian theca cells, causing increased androgen production (Lőrincz et al., 2024). Supporting these mechanisms, UA treatment significantly reduced circulating LH and testosterone levels, achieving results similar to standard medications at a dose of 50 mg/kg (Besasie et al. 2024). The drop in LH levels after UA treatment may indicate some recovery of the hypothalamic-pituitary control, which is often impaired in PCOS. Additionally, the noted hormonal improvements may be partly driven by UA antioxidant properties, considering the link between oxidative stress, neuroendocrine dysfunction, and hormone production in PCOS. The drop in testosterone levels aligns with strong evidence identifying abnormal theca cell function as a key factor in PCOS-related excess androgen levels (Rosenfield & Ehrmann, 2016). However, further detailed investigations are needed to clarify the specific molecular pathways through which UA influences the hypothalamic-pituitary-ovarian axis.

### Quantitative real time PCR

To conduct quantitative real-time PCR, ovarian total RNA was isolated using the EasyRNATM Cell/Tissue RNA Mini Kit (Biovision #K1337). First-strand cDNA was synthesized using Superscript Reverse Transcriptase obtained from Thermo Scientific according to the manufacturer’s protocol (AbdelRahman et al. 2024). Quantitative PCR was performed using an ABI Prism StepOnePlus Real-Time PCR System (Applied Biosystems) with PowerTrackTM SYBR Green Master Mix following the manufacturer’s guidelines. The program used was 95^o^ C for 3 min followed by 40 cycles of denaturation at 94 ^o^ C for 30 s, annealing at 60 ^o^ C for 30 s and extension at 72 ^o^ C for 30 s. Table 1 lists the primer sets for the genes analyzed, and the expression of the target mRNA was normalized to ACTB (Abdel-Gawad et al. 2024). The data were analyzed using the fº method (Gamal and Ibrahim 2024).

**Table 1:** Primer sets of the genes evaluated in the present investigation

	Sense	Antisense	Amplicon	Accession no	Reference
cyp17a1	ACT GAG GGT ATC GTG GAT GC	CCG TCA GGC TGG AGA TAG AC	187	NM_012753.3	(Aziz et al. 2024)
cyp19a1	TGACGTCACTGACAACTCGG	CAAGTCCACGACAGGCTGAT	235	NM_017085.2	(Aziz et al. 2024)
nrf-2	TGTAGATGACCATGAGTCGC	TCCTGCCAAACTTGCTCCAT	159	NM_031789.2	(Mo et al. 2022)
hsd-3b	CTCACATGTCCTACCCAGGC	TATTTTTGAGGGCCGCAAGT	362	NM_001007719.3	(Noshy et al. 2022)
Actb	CCGCGAGTACAACCTTCTTG	CAGTTGGTGACAATGCCGTG	297	NM_031144.3	(Hassanen et al. 2019)

### Histopathological examination

Ovaries were dissected and placed in 10% neutral buffered formalin. Ascending concentration of ethanol and xylene were used for processing of tissues which were then embedded in paraffin wax. A rotary microtome was used for sectioning of tissue into 4 μm thick sections for staining by hematoxylin and eosin stain. A light microscope equipped with digital camera was used for examination.

### Caspase-3 immunohistochemistry

After deparaffinization and rehydration, tissue sections were placed in citrate buffer PH 6 for antigen retrieval. Primary antibodies for caspase-3 (Abexxa, UK) were placed on tissue sections overnight. Anti-species secondary antibodies and substrate were then applied according to manufacturer’s protocol (Bio SB, USA). The percentage of caspase-3 positive expression was estimated by Image J software in three micrographs at 200 X /rat.

### Statistical analysis

The obtained data were analyzed using SPSS software (version 18 for Windows, SPSS Inc., Chicago, IL, USA). Data were analyzed statistically by one-way analysis of variance (ANOVA).

Treatment means were compared by the least significant difference (LSD) at a 5% level of probability, and comparison of means was carried out by Duncan’s Multiple Range Test. Data are presented as the mean ± standard error of the mean (SEM).

## Data Availability

The authors declare that the data supporting the findings of this study are available within the paper.
